# *Leontodon albanicus* subsp. *acroceraunicus* (Asteraceae, Cichorieae): A New Subspecies from Southern Albania

**DOI:** 10.3390/biology14030259

**Published:** 2025-03-04

**Authors:** Fabio Conti, Luca Bracchetti, Marco Dorfner, Nadine Benda, Christoph Oberprieler

**Affiliations:** 1 Scuola di Bioscienze e Medicina Veterinaria, Università di Camerino—Centro Ricerche Floristiche dell’Appennino, Parco Nazionale del Gran Sasso e Monti della Laga, S. Colombo, I-67021 Barisciano, Italy; 2 Scuola di Bioscienze e Medicina Veterinaria, Unità di Ricerca e Didattica di San Benedetto del Tronto (URDIS), Università di Camerino, Via A. Scipioni 6, I-60074 San Benedetto del Tronto, Italy; luca.bracchetti@unicam.it; 3 Evolutionary and Systematic Botany Group, Institute of Plant Biology, University of Regensburg, Universitätsstr. 31, D-93053 Regensburg, Germany; marco.dorfner@ur.de (M.D.); nadine.benda@stud.uni-regensburg.de (N.B.); christoph.oberprieler@ur.de (C.O.)

**Keywords:** AFLPseq fingerprinting, endemic, integrated taxonomy, vascular plants

## Abstract

Some plants belonging to the *Leontodon* sect. *Asterothrix* were collected from southern Albania. They were compared with the closest taxon (*L. albanicus* s.str.) from morphological and molecular (AFLPseq fingerprinting) points of view. Uni- and multivariate statistical analyses of morphological data revealed distinctive discontinuities—especially in terms of the characteristics of the indumentum–that are paralleled by separation into two genetic clusters in AFLPseq fingerprinting. Following an integrated taxonomic approach based on morphological, genetic, and geographical sources of evidence, we show that the newly discovered population should be regarded as a new subspecies named *Leontodon albanicus* subsp. *acroceraunicus*. The new taxon is described and illustrated, and its relationship with *L. albanicus* subsp. *albanicus* is also discussed. We have no data to assess conservation status according to IUCN categories and criteria; however, considering that it is probably limited to the Acroceraunian Mountains, it deserves particular conservation interest.

## Simple Summary

A population of *Leontodon* found in southern Albania on Mt. Ҫika was analyzed from a morphological and genetic point of view. The results allowed us to confirm the distinctness of this population compared to the closest plant species named *L. albanicus*, thus attributing to it a new name: *L. albanicus* subsp. *Acroceraunicus*; it is exclusively found at Mt. Ҫika (S Albania).

## 1. Introduction

*Leontodon* L. (Asteraceae, Cichorieae) includes c. 50 species that are distributed mostly in Europe and the Mediterranean region [1]. During several journeys to the Balkan Peninsula, especially to Croatia (Biokovo Mts.), Albania (Mt. Ҫika and Mt. Nëmerçkë), and Greece (Mt. Tzoumerka, Mt. Kaliakouda, and Mt. Chelidon), we collected some interesting specimens of *Leontodon* belonging to the *Leontodon* sect. *Asterothrix* (Cass. in Cuvier) Ball in order to contribute to the knowledge of this taxonomically critical group. The section *Asterothrix* occurs throughout the Mediterranean region, from the Iberian Peninsula to southwestern Asia [2]. It is usually characterized by three to several-fid or -stellate hairs on the leaves and phyllaries and by all achenes being furnished with pappus bristles. According to Zidorn [3], the section *Asterothrix* is the genus’s most complex group; particularly, the taxonomic structuring of *L. crispus* Vill. *s.lat.* is controversial. Samuel et al. [4] analyzed the genus from a molecular phylogenetic point of view and confirmed the autonomy as the sect. *Asterothrix.* Nine taxa belonging to the genus *Leontodon* are presently recognized in Albania according to POWO [5]: *Leontodon albanicus* (F.K.Mey.) F.Conti, *L. biscutellifolius* DC., *L. crispus* Vill., *L. hispidus* L. subsp. *hispidus*, *L. hispidus* subsp. *hastilis* (L.) Gremli, *L. incanus* (L.) Schrank, *L. rothii* Ball, *L. saxatilis* Lam. subsp. *saxatilis*, and *L. tuberosus* L. *Leontodon incanus* has been reported by mistake for Albania and has to be replaced by *L. albanicus* [6].

In light of the new collections carried out by the first author, some isolated populations have been found that require further investigations. One of these has been collected in southern Albania; here, it is analyzed from a morphological and molecular point of view. It shows peculiar feature combinations and both morphological and genetic distinctness from *L. albanicus* s.str., so we describe it as a subspecies new to science named *L. albanicus* subsp. *acroceraunicus*.

## 2. Materials and Methods

The present study is based on field surveys, an extensive analysis of the relevant literature, and the examination of herbarium specimens of *L. albanicus* and of the new population found, with these kept in APP, FI, and RO (acronyms follow Index Herbariorum [7]).

### 2.1. Morphometric Analyses

The morphological characteristics, recognized as taxonomically important in *Leontodon* (see Table 1), were observed and measured under a stereoscopic microscope using a digital caliper with 0.1 mm precision.

A total of 27 morphological characters were selected and scored for 34 dried individuals. Among the variables examined, 19 were quantitative continuous, and eight were quantitative discrete (Table 1).

The morphometric statistical analysis comprised (as a first step) the evaluation of our data in terms of any departure from normality, which was carried out using the Kolmogorov-Smirnov test [8]. In order to perform comparisons of the two considered groups, the normality test was followed by a Student’s t two independent sample parametric test (for variables following a normal distribution) [9] or a Mann-Whitney U non-parametric test (for non-normally distributed variables). The data were then subjected to a multivariate analysis using Spearman Principal Component Analysis [10,11]. For more information about the differences between the groups studied, the mean and median values, percentiles, and standard deviations were calculated, and box plots were generated. These analyses were carried out with the software program XLSTAT version 2024.4 [12].

### 2.2. AFLPseq Fingerprinting

The recently described AFLPseq fingerprinting procedure based on Oxford Nanopore sequencing technology (ONT; [13]) was used to evaluate genetic variation among nine Albanian herbarium accessions of *L. albanicus* (see Table A1 for further information on genotyped herbarium specimens). Genomic DNA for genetic fingerprinting was extracted following the CTAB DNA extraction protocol of Doyle & Dickson [14] and Doyle & Doyle [15]. The AFLPseq procedure was performed in accordance with the protocol given in Dorfner et al. [13] and Conti et al. [16,17] with the following modifications: in the restriction-ligation step, we used a double-digestion procedure with restriction enzymes MseI and EcoRI. After ligation of MseI and EcoRI adapters (MseI adapter: 5′-GACGATGAGTCCTGAG-3′ + 5′-TACTCAGGACTCAT-3′; EcoRI adapter: 5′-CTCGTAGACTGCGTACC-3′ + 5′-AATTGGTACGCAGTCTAC-3′), we continued with the AFLP genome-reduction protocol using primers with 1bp overhangs (MseI-C: 5′-GATGAGTCCTGAGTAAC-3′; EcoRI-A: 5′-GACTGCGTACCAATTCA) in the pre-selective amplification step; in the selective amplification step, we used additional 1bp- (EcoRI side) or 2bp overhangs (MseI side), respectively. The two primers used in the latter amplification step, however, also included Nanopore barcode adapter sequences at their 5′ ends (Mse_CAA_Nanopore_fw: 5′-TTTCTGTTGGTGCTGATATTGCGATGAGTCCTGAGTAACAA-3′; Eco_AC_Nanopore_rv: 5′-ACTTGCCTGTCGCTCTATCTTCGACTCCGTACCAATTCAC-3′), as suggested in the ‘Ligation sequencing amplicons—PCR barcoding (SQK-LSK114 with EXP-PBC096)’ protocol by Oxford Nanopore Technologies, substituting a subsequent ligation of the Nanopore barcode adapter with an additional barcoding PCR. To ensure specific binding with long and tailed primers, a two-step variation of the selective PCR was conducted (94 °C for 2 min; followed by 30 cycles of 94 °C for 20 s and 72 °C for 2 min; and a final step at 72 °C for 2 min). To every 2 µL of the 1:10 diluted preselective PCR product, 5 µL Taq DNA Polymerase Master Mix RED, 0.25 µL of each 10 µM tailed selective primer, and 2.5 µL H2O were added. After the selective PCR, the length of the fragments ranged from 200 to 700 bp. All subsequent steps (Nanopore barcode PCR, sample multiplexing, size selection, and preparation of Nanopore sequencing library) followed Dorfner et al. [13]. The resulting library was sequenced with a MinION using a Flongle flow cell (R10.4.1, FLO-FLG114). Read data processing, de novo locus assembly, identification of orthologous loci, reference-based SNP calling with the SLANG pipeline, and the final calculation of Jukes-Cantor (JC) distances were carried out according to the protocol described by Dorfner et al. [13] and Conti et al. [17]. Based on these pairwise distances, a principal co-ordinate analysis (PCoA) with a custom R v.4.0.5 script using the ‘phangorn‘ library to read the distance matrices and the ‘ape‘ package to calculate and plot the PCoA was carried out.

## 3. Results

### 3.1. Morphometric Analyses

The variability of the analyzed morphological characters was described using standard statistical parameters. In the taxonomic treatment, the values in brackets express the minimum and maximum values observed, while the given intervals represent the 25th and 75th percentiles. For a comparison of the means between the two considered groups (*L. albanicus* s.str. and the newly investigated population from Mt. Ҫika), Student’s t and Mann-Whitney U tests were carried out on the basis of the departure from the normality distribution of the data variables; Table 1 shows the obtained *p*-values for each variable.

**Figure 1:**
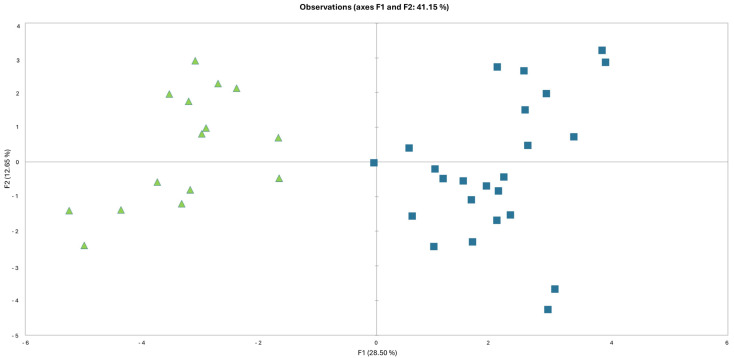
Scatter plot of Principal Component Analysis; accessions of the Ҫika population are on the left side (green triangles); accessions of the Nëmerçkë population of *L. albanicus* are on the right side (blue squares).

To verify the existence of a pattern that defines the morphological distinctness of the two studied entities, Principal Component Analysis (PCA) was performed. Based on the arrangement of the first two principal components (28.5% and 12.65% of the total variance, respectively), a good separation of the Nemërçkë and Mt. Ҫika populations was observed (Figure 1).

The variables that contribute the highest loadings to the first two principal components are the following: Leaf hairs: the most common number of distal rays (11.1%), the minimum number of distal rays (9.1%), and the maximum number of distal rays (9.4%); involucre: external bract length (mm) (7.6%); stellate hairs on the external involucral bracts: the number of rays (7.3%); stellate hairs on the middle involucral bracts: the number of rays (6.6%); pappus: ray length (mm) (5.9%). Variation along the second principal co-ordinate is mostly explained by plant height (12.3%), leaf length (13.7%), leaf width (11.2%), stellate hairs on the external involucral bract margins, and stalk length (10.6%).

### 3.2. AFLPseq Fingerprinting

**Figure 2:**
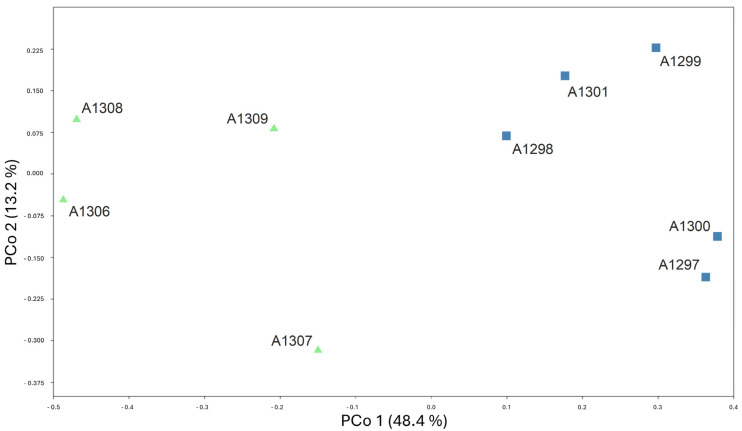
Ordination of nine accessions of *Leontodon albanicus* on the first two axes of Principal Co-ordinate Analysis (PCoA) based on 9260 parsimony informative SNPs from AFLPseq fingerprinting with Jukes-Cantor distances as a measure of genetic similarity among accessions. Ҫika population is on the left (green triangles); Nëmerçkë population of *L. albanicus* is on the right (blue squares).

In total, 104,553 reads and 27.08 Mbp were sequenced for the nine Leontodon accessions. After read pre-processing, 102,703 reads with lengths between 50 bp and 700 bp passed the Q5 quality filter. With the SLANG pipeline (cluster thresholds set to 0.85 for the first and second cluster step), 9260 parsimony informative SNPs were mined from orthologous loci. After the calculation of pairwise JC distances (see Supplementary Materials), the resulting PCoA plot was obtained (Figure 2). The PCoA plot demonstrates the clear separation between the two morphologically defined taxa; accessions of the Mt. Ҫika population are on the left side, and accessions of the Nemërçkë populations of *L. albanicus* are on the right side of principal co-ordinate PCo axis 1, which accounts for 48.4% of the total variance in the dataset; PCo axis 2 accounts for only an additional 13.2% of the total variance. The genetic analysis, therefore, is in complete correspondence with the results of the morphological data and supports the treatment of the Mt. Ҫika populations as an independent taxonomic unit. In the following, due to the allopatric distribution of the two entities, acknowledgment at subspecies level is suggested following the line of reasoning of Oberprieler [18].

## 4. Discussion

The new subspecies found on Mt. Ҫika is similar to *L. albanicus* s.str. but can be distinguished by several morphological characters, as shown in the results and in the diagnosis. The two taxa both live in southern Albania but in different territories. *Leontodon albanicus* subsp. *albanicus* occurs in the Gjirokastër district (Rrethi i Gjirokastrës) near Fushë Bardhë and Mt. Nëmerçkë (even in the Greek portion of the mountain); furthermore, it is recorded here for Maja e Këndrevicës. *Leontodon albanicus* subsp. *acroceraunicus* is known only for Mt. Ҫika. At the moment, the complete distribution range and the number of individuals are not known; it is possible that, in addition to the summit, it could be present elsewhere in the impervious Acroceraunian Mountains. Therefore, at the moment, we have no data to assess the conservation status according to IUCN categories and criteria; however, considering that it is probably limited to the Acroceraunian Mountains, it deserves particular conservation interest.

Reports by Baldacci [19] of *L. graecus* “*in lapidosis m. Cika*” using collection number 149 correspond perfectly with the observed specimens in FI and RO (see *specimina visa*) for the same locality and number, and they are to be referred to as *L. albanicus* subsp. *acroceraunicus*.

The Acroceraunian Mountains are bordered by the sea to the southwest and isolated from the other mountain groups by low valleys. They reach an altitude of 2044 m with Mount Ҫika. The isolation and altitude have allowed the differentiation of this taxon and another strictly endemic plant for the Acroceraunian Mountains, such as *Reichardia albanica* F. Conti & D. Lakušić. The new taxon is added to the 4004 plants recorded for Albania [20].

## 5. Conclusions

The morphological and molecular analyses provide evidence that the new population found on Mt. Ҫika should be regarded as a new subspecies endemic to the Acroceraunian Mountains (Southern Albania) (see taxonomic treatment).

### Taxonomic Treatment

**Figure 3:**
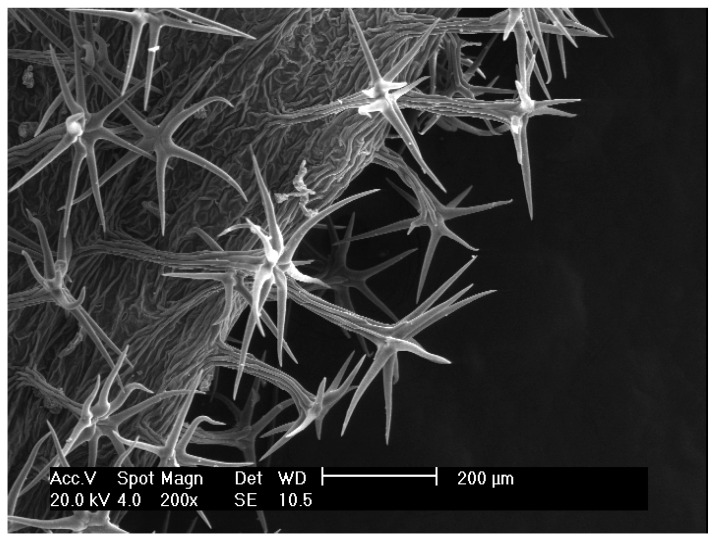
Leaf hairs from a specimen of *L. albanicus* subsp. *albanicus* collected on Mt. Nëmerçkë.

***Leontodon albanicus*** (F.K.Mey.) F.Conti in Phytotaxa 360: 288 (2018) subsp. ***albanicus*** (Figure 3).

Holotype (Conti 2018: 288):—ALBANIA. Mali i Gjer (Mali Gjinezh), zwischen Qafa Gradishtit und Qafa Piloit, ca. 1400 m, 26 June 1959, *F. K. Meyer 3397* (JE, [digital image]!).

Perennial, with a taproot and a single unbranched stem, (76–)98–153(–280) mm tall. Stem ribbed, with shortly stalked, apically 5–9-fid hairs; bracts: 0–3. Basal leaves rosulate: (31–)51.8–76.3(–125) × (4–)9–15(–20) mm, oblanceolate, and sinuate-dentate, with (1–)3–4(–7) teeth; teeth width: (0.3–)1.3–2.0(–4.2) mm, greyish-green, with dense [number of hairs per 1 mm^2^: (15.0–)26.0–34.3(–47.0)], stalked, usually (6–)8-fid hairs on both surfaces: minimum: (4–)5–6, maximum: (8–)9–10(–11)-fid hairs; stalk length: (0.10–)0.14–0.19(–0.27) mm; maximum stalk length: (0.14–)0.22–0.30(–0.37) mm; ray length: (0.13–)0.16–0.22(–0.28) mm. Capitulum solitary. Involucre length: (12–)13–15(–19) mm, phyllaries linear-lanceolate, in several rows: outer: (3.5–)5.0–6.0(–7.0) mm long, with flexuose stalked (3–)5(–8)-fid hairs, (0.9–)1.2–1.8(–3.0) mm long on the surface, with moniliformous hairs (0.10–)0.18–0.23(–0.38) mm long; marginally pectinate-ciliate with stellate hairs (3–)5(–8)-fid, stalk: (0.0–)0.05–0.8(–2.0) mm long; rays: (0.10–)0.13–0.28(–0.50) mm long. Middle bracts surface stellate hairs (0–)3–5-fid; stalk: (0–)0.55–1.3(–2.3) mm long, rays: (0–)0.18–0.25(–0.8) mm long. Ligules yellow: (13.0–)18.8–25.0 mm long. Achene: (4.0–)4.8–5.5(–6.3) mm, with triangular scale-like above, narrowed towards the apex. Pappus off-white: (7.3–)9.0–10.1(–11.5) mm: scabrid, with few longer hairs: (0.0)0.20–0.36(–0.48) mm, denticules: (0.03–)0.04–0.06(–0.8) mm.

Flowering from late May to the beginning of July; fruiting from the end of June to July.

**Figure 4:**
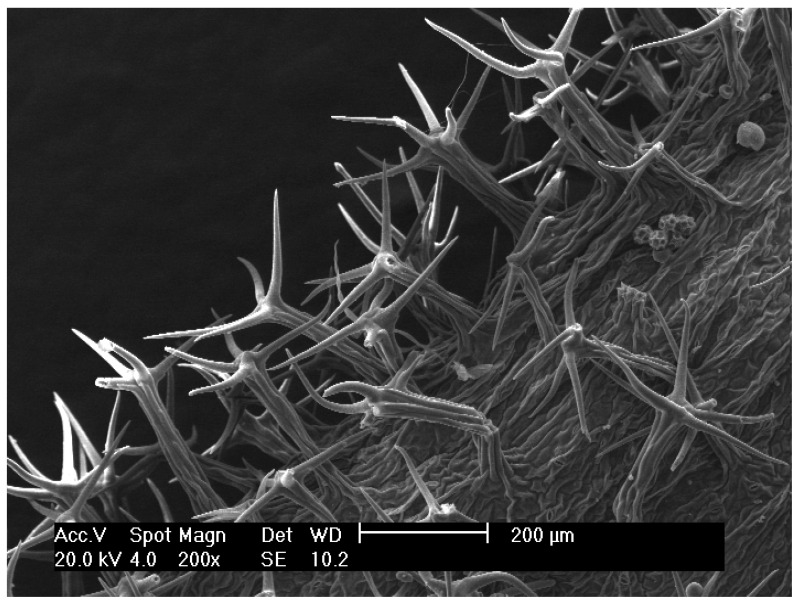
Leaf hairs from a specimen of *L. albanicus* subsp. acroceraunicus collected on Mt. Ҫika.

**Figure 5:**
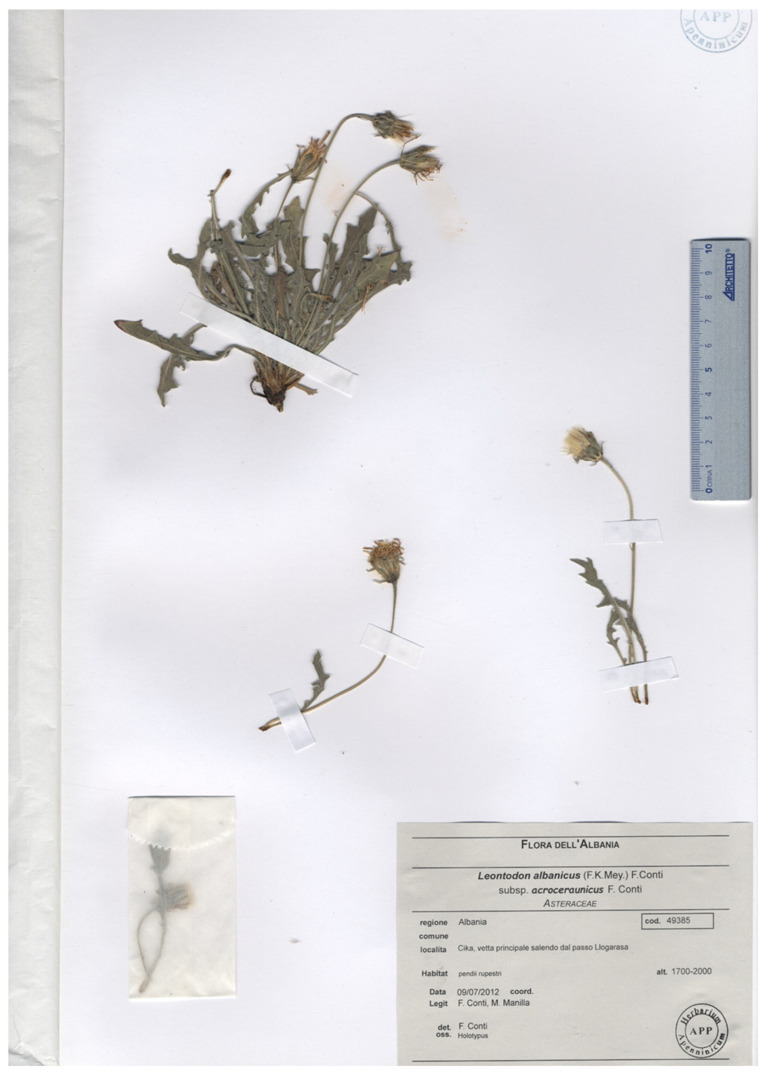
Holotypus of *Leontodon albanicus* subsp. *acroceraunicus*.

***Leontodon albanicus*** (F.K.Mey.) F.Conti subsp. ***acroceraunicus*** F.Conti, **subsp. nov.** (Figure 4 and Figure 5).

Holotype:—Albania. Ҫika, vetta principale salendo dal passo Llogarasa 1700–2000 m, 09/07/2012, *F. Conti*, *M. Manilla* (APP No. 49385) Isotype (APP No. 49368).

Diagnosis: It is distinguished from *Leontodon albanicus* subsp. *albanicus* by stems with shortly stalked, apically (3–)4(–5)-fid hairs vs. 5–9-fid hairs; leaves with stalked and usually 4–(5)-fid [from minimally 3–4- to maximally 5–6(–8)-fid] hairs on both surfaces vs. leaves with stalked and usually (6–)8-fid [from minimally (4–)5–6- to maximally (8–)9–10(–11)-fid] hairs on both surfaces; involucres with shorter external bracts: (2.5–)3.0–4.0(–5.5) mm vs. (3.5–)5.0–6.0(–7.0) mm long surface for middle bracts with stellate hairs (1–)2–3-fid vs. (0–)3–5-fid; pappus: (5.9–)7.0–8.8(–11.0) mm vs. (7.3–)9.0–10.1(–11.5) mm.

Description: Perennial, with a taproot and 1 unbranched stem, (65–)80–142(–170) mm tall. Stem ribbed, with short-stalked (3–)4(–5)-fid hairs; bracts: 0–3. Basal leaves rosulate: (22.0–)42.5–67.0(–95.0) × (5.0–)6.5–10.5(–13) mm, oblanceolate, sinuate-dentate, with (2–)3–5(–7) teeth; teeth width: (0.8–)1.1–3.0(–7.0) mm, greyish-green, with dense (number of hairs in 1 mm^2^) (31–)35–52(–95), stalked (usually 4(–5)-fid) hairs on both surfaces: min.: 3–4-, max.: 5–6(–8)-fid hairs; stalk length: (0.06–)0.13–0.25(–0.32) mm; max stalk length: (0.16–)0.26–0.46(–0.55) mm; ray length: (0.12–)0.2–0.24(–0.27) mm. Capitulum solitary. Involucre length: 10–14(–16) mm; phyllaries: linear-lanceolate, in several rows: outer: (2.5–)3.0–4.0(–5.5) mm long, with flexuous, stalked (1–)2.2–3(–4)-fid hairs, (0.83–)1.12–1.47(–2.0) mm long on the surface, with moniliforms hairs (0.12–)0.15–0.2(–0.31) mm long, marginally pectinate-ciliate with simple or stellate hairs (1–)2–3.7(–5)-fid; stalk: (0.06–)0.08–0.18(–0.34) mm long; rays: (0–)0.04–0.16(–0.23) mm long. Middle bracts surface stellate hairs: (1–)2–3-fid; stalk: (0.18–)0.82–1.45(–2.2) mm long; rays: (0–)0.1–0.49(–1.1) mm long. Ligules yellow: (12–)13.1–15.4(–20) mm long. Achene: (3.5–)4.2–5.9(–6) mm with triangular, scale-like above, narrowed towards the apex. Pappus off-white: (5.9–)7.0–8.8(–11) mm, scabrid with few longer hairs: (0–)0.2–0.4(–0.55) mm; denticules: (0.02–)0.03–0.05(–0.07) mm. Flowering in June to the beginning of July and fruiting in July.

Etymology: The specific epithet refers to the Acroceraunian Mountains, where this subspecies of *Leotodon albanicus* is found.

**Figure 6:**
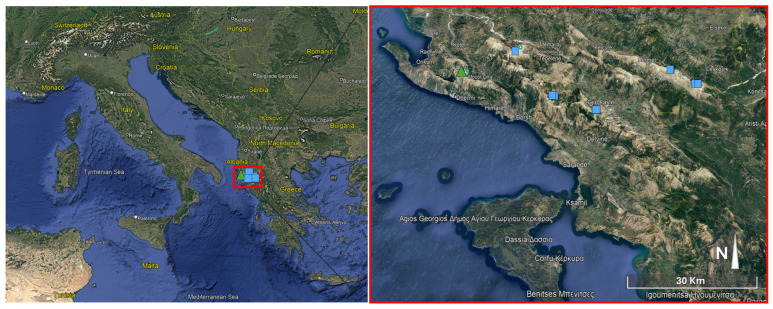
Distribution map of *Leontodon albanicus* according to the herbarium materials studied: subsp. *albanicus* (blue squares); subsp. *acroceraunicus* (green triangles).

Distribution and habitat: It is known from southern Albania on Mt. Ҫika (Acroceraunian mountains) (Figure 6).

**Specimina visa** (for *L. albanicus* subsp. *albanicus*, only the specimens that expand the known distribution area [6] are added):

***L. albanicus* subsp. *albanicus* Albania:** Vlorë, Vranisht, Maja e Këndrevicës, consolidates screes and grasslands with sparse shrubs, limestones, 1100–1700 m a.s.l., 8 July 2020, *E. Ammann*, *L. Caucci*, *E. De Luca*, *F. Buttarazzi*, *F. Guidantoni* (RO Nos. 30334, 30376);

***L. albanicus* subsp. *acroceraunicus*. Albania**: in lapidosis Mt. Ҫika (Acroceraunia), 13 July 1892, *A. Baldacci 149*, as *L. graecum* (FI, RO); Vlorë, Vranisht, Maja Ҫikes, rock vegetation with *Saxifraga* spp., limestone, 40.20083° N 19.632881° E 1796 a.s.l., 4 July 2020, *S. De Sanctis s.n*. (RO No. 30190); Vlorë, Vranisht, Maja Ҫikes, *Saxifraga* rock vegetation, limestone, 40.20397° N 19.628791° E, 1818 a.s.l., 4 July 2020, *S. De Sanctis s.n.* (RO); Vlorë, Horë-Vranisht, Ҫika, limestone, 2016–2045 m a.s.l., 5 July 2020, *L. Caucci*, *S. De Sanctis*, *E. Ammann*, *T. Ammann*, *C.Ammann*, *E. De Luca*, *C. Alessandrini*, *S. Frasca*, *F. Buttarazzi* (RO Nos. 33619, 33614).

## Figures and Tables

**Table 1:** Descriptive statistics and comparison significance (group 1 vs. group 2) for each of the 27 considered variables. To assess the difference between the two groups for each surveyed variable, Student’s *t* tests (*) and Mann-Whitney U tests (**) were used; the statistically significant differences (<0.05) are given in bold.

	Group 1 (*L. albanicus* subsp. *albanicus*)			Group 2 (*L. albanicus* subsp. *acroceraunicus*)			
Character	Mean	Min	Max	Mean	Min	Max	*p*-Value
Plant height (cm) *	132.17	76	280	110.28	65	170	0.162
Leaf length (mm) *	64.62	31	125	53.47	22	95	0.121
Leaf width (mm) *	11.67	4	20	8.67	5	13	0.011
Number of teeth along each leaf side **	3.21	1	7	3.87	2	7	0.144
Width of leaf teeth (mm) **	1.77	0.3	4.2	2.41	0.8	7	0.265
Number of hairs per 1 mm^2^ on leaf surface *	31.33	15	47	47.38	31	95	0.001
Leaf hairs: most common number of distal rays **	7.71	6	8	4.13	4	5	<0.001
Leaf hairs: minimum number of distal rays **	5.29	4	6	3.33	3	4	<0.001
Leaf hairs: maximum number of distal rays **	9.33	8	11	5.8	5	8	<0.001
Leaf hairs: stalk length (mm) *	0.17	0.1	0.27	0.19	0.06	0.32	0.269
Leaf hairs: maximum stalk length (mm) *	0.26	0.14	0.37	0.34	0.16	0.55	0.006
Leaf hairs: ray length (mm) *	0.19	0.13	0.18	0.21	0.12	0.27	0.158
Involucre: length (mm) *	14.33	12	19	12.15	10	16	0.002
Involucre: external bract length (mm) *	5.43	3.5	7	3.71	2.5	5.5	<0.001
Stellate hairs on external involucral bracts: number of rays **	4.95	3	8	2.78	1	4	<0.001
Hairs on external involucral bracts: hair length (mm) *	1.58	0.9	3	1.35	0.83	2	0.113
Hairs on external and/or middle involucral bracts: length of simple moniliformis hairs (mm) *	0.2	0.1	0.38	0.18	0.12	0.31	0.251
Stellate hairs on external involucral bract margins: number of rays **	5.05	3	8	2.71	1	5	<0.001
Stellate hairs on external involucral bract margins: stalk length (mm) **	0.46	0	2	0.13	0.06	0.34	0.129
Stellate hairs on external involucral bract margins: ray length (mm) *	0.21	0.1	0.5	0.1	0	0.23	0.001
Stellate hairs on middle involucral bract: number of rays *	4.05	0	5	2.21	1	3	<0.001
Stellate hairs on middle involucral bract: stalk length (mm) *	1.05	0	2.3	1.13	0.18	2.2	0.698
Stellate hairs on middle involucral bract: ray length (mm) **	0.26	0	0.8	0.3	0	1.1	0.913
Achene: length (mm) *	5.19	4	6.3	4.9	3.5	6	0.152
Pappus: ray length (mm) *	9.38	7.3	11.5	8,05	5.9	11	0.001
Pappus: plume length (mm) *	0.28	0	0.48	0.3	0	0.55	0.52
Pappus: denticule length (mm) *	0.05	0.03	0.08	0.04	0.02	0.07	0.003

## Data Availability

The data presented in the current study are available within the article. Raw data of the AFLPseq fingerprinting were submitted to GenBank under the BioProject accession number PRJNA1217834.

## Supplementary Materials

The following supporting information can be downloaded at: https://www.mdpi.com/article/10.3390/biology14030259/s1, Table S1: Pairwise Jukes-Cantor (JC) distances among *Leontodon albanicus* accessions of the present study based on 9260 parsimony informative SNPs from an AFLPseq fingerprinting analysis.

## Appendix A

**Table A1:** *Leontodon* specimens molecularly analyzed in the present study with information on localities and voucher specimens.

Samples	Taxon	Locality	Collectors	Voucher Specimens
A1297	*albanicus*	ALB, Nemërçkë, Maja e Papingut, 2180 m	Conti & Manilla s.n.	APP 48886
A1298	*albanicus*	ALB, Nemërçkë, 500–120 m	Conti et al. s.n.	APP 57114
A1299	*albanicus*	ALB, Nemërçkë, 500–120 m	Conti et al. s.n.	APP 57115
A1300	*albanicus*	ALB, Nemercka—Diopala.	Lakušić et al. s.n.	APP 56301
A1301	*albanicus*	ALB, Poliçan—Nemërçkë, 1200–2480 m	Conti et al. s.n.	APP 56996
A1306	*acroceraunicus*	ALB, Llogarasa pass, 1700–2000 m	Conti & Manilla s.n.	APP 49368
A1307	*acroceraunicus*	ALB, Llogarasa pass, 1700–2000 m	Conti & Manilla s.n.	APP 49368
A1308	*acroceraunicus*	ALB, Llogarasa pass, 1700–2000 m	Conti & Manilla s.n.	APP 49368
A1309	*acroceraunicus*	ALB, Llogarasa pass, 1700–2000 m	Conti & Manilla s.n.	APP 49385
