# Supplementary material for: Leontodon albanicus subsp. acroceraunicus (Asteraceae, Cichorieae): A New Subspecies from Southern Albania
Source: Biology (Basel). 2025 Mar 4;14(3):259. doi: 10.3390/biology14030259 (PMC11940545; doi:10.3390/biology14030259)
Supplement: Supplementary file 1 [file biology-14-00259-s001.zip › biology-3487705-supplementary.pdf]

Table S1. Pairwise Jukes-Cantor (JC) distances among *Leontodon albanicus* accessions of the present study based on 9260 parsimony informative SNPs from an AFLPseq fingerprinting analysis.

	**A1309**	**A1298**	**A1300**	**A1297**	**A1307**	**A1308**	**A1301**	**A1299**
**A1309**	0.000							
**A1298**	0.397							
**A1300**	0.526	0.302						
**A1297**	0.522	0.191	0.152					
**A1307**	0.265	0.331	0.350	0.355				
**A1308**	0.172	0.511	0.760	0.825	0.203			
**A1301**	0.417	0.087	0.230	0.176	0.441	0.567		
**A1299**	0.439	0.171	0.135	0.222	0.506	0.682	0.098	
**A1306**	0.204	0.468	0.842	0.749	0.132	0.045	0.576	0.737
